# Health Information–Seeking Behaviors of Family Caregivers: Analysis of the Health Information National Trends Survey

**DOI:** 10.2196/11237

**Published:** 2019-01-14

**Authors:** Lauren R Bangerter, Joan Griffin, Kristin Harden, Lila J Rutten

**Affiliations:** 1 Robert D and Patricia E Kern Center for the Science of Healthcare Delivery Mayo Clinic Rochester, MN United States

**Keywords:** disparities, family caregivers, Health Information National Trends Survey, internet use, mobile phone

## Abstract

**Background:**

The growing population of aging adults relies on informal caregivers to help meet their health care needs, get help with decision making, and gather health information.

**Objective:**

The objective of this study was to examine health information–seeking behaviors among caregivers and to identify caregiver characteristics that contribute to difficulty in seeking health information.

**Methods:**

Data from the Health Information National Trends Survey 5, Cycle 1 (N=3181) were used to compare health information seeking of caregivers (n=391) with noncaregivers (n=2790).

**Results:**

Caregivers sought health information for themselves and others using computers, smartphones, or other electronic means more frequently than noncaregivers. Caregivers born outside of the United States reported greater difficulty seeking health information (beta=.42; *P*=.02). Nonwhite caregivers (beta =−.33; *P*=.03), those with less education (beta =−.35; *P*=.02), those with private insurance (beta =−.37; *P*=.01), and those without a regular health care provider (beta =−.35; *P*=.01) had less confidence seeking health information. Caregivers with higher income had more confidence (beta =.12; *P*≤.001) seeking health information.

**Conclusions:**

This study highlights the prevalence of electronic means to find health information among caregivers. Notable differences in difficulty and confidence in health information seeking exist between caregivers, indicating the need for more attention to the socioeconomic status and caregivers born outside of the United States. Findings can guide efforts to optimize caregivers’ health information–seeking experiences.

## Introduction

Family caregivers play a critical role in supporting the health, well-being, and quality of life for patients with chronic health conditions. Caregivers are defined as unpaid family members or friends who provide support and care to a loved one with a chronic health condition. Caregivers assist with activities of daily living, such as bathing, dressing, and toileting, provide emotional and social support, manage medications and finances, communicate with health care providers, and advocate on behalf of their loved one. Thus, caregivers are among the most engaged of health care stakeholders. Caregiving requires heightened awareness and knowledge of specific health information, medications, cost, insurance, health conditions, and the health care system. As such, family caregivers are often highly engaged in the pursuit of health information, support, and advice, both for themselves and others, more frequently than noncaregivers [1,2]. Caregivers rely on a range of informational sources, settings, and technologies, including referrals to other health care professionals, print material, community resources, and Web-based sources [3].

The availability and widespread use of the internet have increased the quantity of available health information and the speed at which caregivers can obtain resources and information. Research has found that caregivers use the internet to seek general information on the medical, emotional, and financial aspects of caregiving, as well as seek assistance in interpreting symptoms, health conditions, and changes in patient behavior and relationships [4]. In addition, caregivers use internet-based peer support communities to seek information and emotional support, relying on the subjective opinions, experiences, and advice of fellow caregivers [5]. With the convergence of an aging population, rising health care costs, and the ubiquitous use of Web-based platforms to distribute health information, engagement of caregivers in health information technology has the potential to advance the clinical quality, caregiver well-being, and patient safety [6]. Furthermore, internet-based interventions are becoming increasingly prominent and provide accessible and affordable opportunities to remotely support caregivers [7]. Indeed, caregiver interventions once delivered in-person are now being successfully adapted into Web-based programs such as the web adaptation of the Savvy Caregiver Program and the FOCUS Program [8-10]. As more internet-based psychoeducational interventions are developed and adapted as Web-based programs, it is critical to continually assess how and why caregivers use the internet, so that interventions and Web-based services can be developed in ways that are congruent to the values, needs, preferences, and practices of caregivers.

To date, research has largely focused on barriers and facilitators of patients’ use of the internet to seek health information, while far less is known about caregivers’ appraisals of health information seeking [11]. As the shift of health information to Web-based platforms becomes more apparent, and the number of family caregivers’ increases, it is critical to pay more attention to how caregivers use the internet to access health information and track disparities in information seeking among caregivers. In addition, studies have reported challenges surrounding health information seeking among patient populations, but caregivers’ appraisal of health information seeking has received less attention [6]. Examining appraisals of health information seeking can yield valuable insight into caregivers who may need more tailored delivery of health information. Likewise, caregivers who are confident in health information seeking may be well positioned to assist in health communication campaigns.

Using a nationally representative sample drawn from the Health Information National Trends Survey (HINTS), this study aims to describe a variation in health information–seeking behaviors between caregivers and noncaregivers and their usage of the internet to find health information.

## Methods

### Sample

HINTS is a nationally representative survey administered by the National Cancer Institute since 2003. The HINTS target population is adults aged ≥18 years in the civilian noninstitutionalized population of the United States. HINTS 5 (Cycle 1) was conducted from January 25, 2017 to May 5, 2017. The questionnaire included a series of questions about caregiving.

The sampling frame consisted of a database of addresses used by the Marketing Systems Group to provide random samples of addresses. The questionnaire was administered as a mailed survey; a total of 4 emails were sent out. All households in the sample received the first mailing and reminder postcard, while only nonresponding households received the subsequent survey mailings. Details on sampling strategies and survey design are available in the HINTS 5, Cycle 1 methodology report [12]. The final weighted response rate was 32.39% (3240/13,360).The final response rate was calculated using the RR2 formula of the American Association of Public Opinion Research.

### Measures

#### Sociodemographic Variables

The following sociodemographic variables were included in analyses: sex (male, female), age (range 18-101 years), race (white, nonwhite) or ethnicity (Hispanic, non-Hispanic), annual household income (treated as a continuous variable; see Table 1 for categories), education (less than high school or high school graduate, some college, college or bachelor’s degree), born in the United States (yes, no), employment status (employed for wages, not employed for wages), home ownership (own, rent, occupy without paying monetary rent), health insurance (yes, no), type of health insurance (employer-provided insurance, insurance purchased from an insurance company, Medicaid, Medicare, TRICARE or Veterans’ Affairs insurance, other insurance), have a regular health care provider (yes, no), and marital status (married or living as married, not married).

#### Caregiver Status

To determine the caregiver status, participants were asked, “Are you currently caring for or making health care decisions for someone with a medical, behavioral, disability, or other condition?” Participants who responded “yes” also indicated whom they provided care to (child, spouse, parent, another family member, or friend). In this study, we excluded participants who indicated they provided care for a child because of the unique differences in responsibilities and experiences between this and other types of caregiving relationships. Participants who self-identified as caregivers were asked to indicate, “All conditions for which you have provided care for this person.” Responses included cancer, Alzheimer’s or confusion or dementia or forgetfulness, orthopedic or musculoskeletal issues, mental health or behavioral or substance issues, chronic conditions, neurological/ developmental issues, acute conditions, aging or aging-related issues, other, and do not know. Furthermore, participants were asked, “Thinking of all of the kinds of help you provide for this person or persons, about how many hours do you spend in an average week providing care?”: (<5 hours/week, 5-14 hours/week, 15-20 hours/week, 21-34 hours/week, and ≥35 hours/week).

#### Health Information Seeking

Participants were asked a series of questions to assess their health information–seeking behavior. Participants were first asked, “Have you ever looked for information about health or medical topics from any source?” Those who answered “yes” were asked, “The most recent time you looked for health information, who was it for (myself, someone else, both)?” and “The most recent time you looked for information about health or medical topics, where did you go first?” Responses were coded as books, brochures, pamphlets, cancer organization, family, friend or coworker, doctor or health care provider, internet, library, magazines, newspapers, telephone information number, and complementary, alternative, or unconventional practitioner.

To assess difficulty seeking health information, participants were asked the extent to which they agree (strongly agree to strongly disagree on a 4-point scale) with a series of statements based on the results of their most recent search for information about health or medical topics (“It took a lot of effort to get the information you needed”; “You felt frustrated during your search for the information”; “You were concerned about the quality of the information”; “The information you found was hard to understand”). Reliability statistics were calculated between these items, and good reliability was found (Cronbach alpha=.85), along with a singular factor structure. As such, a mean score was calculated among the items to create a singular difficulty in seeking health information measure. To assess confidence, all participants were asked “Overall, how confident are you that you could get advice or information about health or medical topics if you needed it?” on a scale ranging from 1 “not confident at all” to 5 “completely confident.”

#### Using the Internet to Find Health Information

Participants were asked a series of yes or no questions to assess their use of the internet to find health information: “In the past 12 months, have you used a computer, smartphone, or other electronic means to do any of the following?” (“Look for health or medical information for yourself”; “Look for health or medical information for someone else”; “Buy medicine or vitamins online”; “Look for a health care provider”; “Use email or the internet to communicate with a doctor or a doctor’s office”; “Made appointments with a health care provider”; “Track health care charges and costs”; “Fill out forms or paperwork related to your health care”; “Look up test results”).

### Data Analysis

Data analysis was performed using SAS/STAT software, Version 8 of the SAS System for Unix. Copyright (2018; SAS Institute Inc). First, univariate analyses were conducted for the sample, including the descriptive statistics only for participants who identified themselves as caregivers (Table 2). Next, bivariate analyses were conducted to examine the relationships between the variables of interest, including comparisons of the measures between caregivers and noncaregivers (Tables 1, 2, and 3). Finally, to understand the experience of caregivers seeking health information, 2 multivariate linear regression models were built using respondents who identified as caregivers—one with the measure of confidence in seeking health information as the dependent variable, and one with the difficulty seeking health information scale measure as the outcome (Table 4); all models were adjusted for. For all analyses, replicate weights were applied to account for the complex sampling design and derive representative estimates.

## Results

### Sociodemographic Characteristics of Caregivers

Table 1 summarizes the descriptive statistics for demographic and key caregiver variables. Caregivers (n=391) comprised 237 (58.4%) females and had an average age of 51.8 (SD 1.60) years. Among the 391 caregivers, 166 (51.4%) were employed, 266 (66.8%) were married, 251 (69.4%) were white, 313 (87.3%) were non-Hispanic, and 327 (84.7%) were born in the United States. The education level of the 391 caregivers was as follows: 98 (31.0%) had a high school education or less, 131 (33.9%) had some post-high school education, and 162 (35.1%) had a college degree or more.

Table 2 summarizes descriptive information on caregiver relationship and activities. Of the 391 caregivers, 148 (41.4%) reported that they provide care for a parent or parents, 92 (20.5%) provided care for a spouse, 50 (10.4%) provided care to another family member, and 24 (4.1%) provided care to a friend or nonrelative. In addition, 239 (63.6%) provided care to a person with multiple conditions and 136 (32.8%) reported providing <5 hours of care per week.

### Caregivers’ Health Information Seeking

Caregivers reported looking for health information (from any source) more than noncaregivers (Table 3). The internet was the most frequently used source of health information. More noncaregivers (1401/1932, 72.52%) reported using the internet in their most recent search for health information compared with caregivers (181/278, 65.1%). Caregivers more frequently reported that the last time they looked for health information was for both themselves and someone else, while most noncaregivers reported looking for health information for themselves only. Caregivers and noncaregivers reported low difficulty in seeking health information and moderate levels of confidence in obtaining information on medical topics.

### Caregivers’ Internet Experience

Among respondents who reported using the internet, caregivers used a computer, smartphone, or electronic means for health information–seeking activities more often than noncaregivers (Table 3). Compared with noncaregivers, caregivers more frequently use the internet to find health information for others (264/391, 71.1%), and make appointments with a health care provider (166/391, 46.8%).

In multivariable models predicting difficulty in health information seeking, we found that caregivers who were born outside of the United States reported greater difficulty seeking health information (beta=.42; *P*=.02; Table 4). In multivariable models predicting caregivers’ confidence in health information seeking, we found that nonwhite caregivers (beta=−.33; *P*=.03), those with less education (beta=−.35; *P*=.02), those with private insurance compared with public insurance (beta=−.37; *P*=.01), and those without a regular health care provider (beta=−.35; *P*=.01) had lower confidence in seeking health information. Caregivers with higher income had greater confidence (beta=.12; *P* ≤.001) in seeking health information (Table 4).

**Table 1:** Descriptive statistics for demographic variables.

Variables		Caregiver (n=391), n (%)	Noncaregiver (n=2790), n (%)	*χ*^2^ (*df*)	*P* value
**Sex**				3.8 (1)	.05
	Female	237 (58.42)	1507 (49.85)	—^a^	—
	Male	128 (41.58)	1097 (50.15)	—	—
Age in years		51.8 (13.93)^b^	46.63 (16.39)^b^	−3.03 (3180)^c^	.003
**Employment status**				4.3 (1)	.04
	Employed	166 (48.64)	1396 (59.76)	—	—
	Not employed	202 (51.36)	1276 (40.24)	—	—
**Marital status**				7.6 (1)	.006
	Married or living as married	266 (66.79)	1547 (54.85)	—	—
	Not married	125 (33.21)	1243 (45.150	—	—
**Education level**				0.2 (2)	.89
	High school graduate or less	98 (31.00)	770 (32.14)	—	—
	Some post-high school education	131(33.93)	789 (32.18)	—	—
	College graduate or more	162 (35.07)	1231 (35.68)	—	—
**Born in the United States**				0.1 (1)	.72
	Yes	327 (84.70)	2364 (85.66)	—	—
	No	58 (15.29)	369 (14.34)	—	—
**Ethnicity**				1.2 (1)	.27
	Hispanic	48 (12.72)	365 (15.82)	—	—
	Non-Hispanic	313 (87.27)	2196 (84.18)	—	—
**Race**				5.1 (1)	.02
	White	251 (69.35)	1924 (77.55)	—	—
	Nonwhite	121 (30.64)	686 (22.45)	—	—
**Home ownership**				5.7 (2)	.06
	Own	286 (73.01)	1915 (62.42)	—	—
	Rent	87 (23.00)	729 (33.58)	—	—
	Occupy without paying monetary rent	7 (3.98)	57 (4.00)	—	—
**Household income (US $)**				7.1 (8)	.52
	0-9999	20 (5.264)	179 (6.84)	—	—
	10,000-14,999	16 (4.76)	157 (4.59)	—	—
	15,000-19,999	31 (8.54)	128 (5.58)	—	—
	20,000-34,999	50 (10.86)	358 (12.46)	—	—
	35,000-49,999	56 (17.24)	321 (14.52)	—	—
	50,000-74,999	71 (18.03)	447 (18.98)	—	—
	75,000-99,999	41 (11.82)	318 (12.33)	—	—
	100,000-199,999	60 (19.94)	456 (17.89)	—	—
	≥200,000	15 (3.50)	157 (6.82)	—	—
**Has a regular health care provider**				1.9 (1)	.17
	No	100 (29.37)	792 (34.94)	—	—
	Yes	289 (70.63)	1968 (65.06)	—	—
**Has health insurance**				0.1 (1)	.76
	No	19 (7.19)	130 (8.16)	—	—
	Yes	370 (92.81)	2630 (91.83)	—	—
**Health insurance type**				14.7 (3)	.002
	Private^d^	159 (40.87)	1227 (44.46)	—	—
	Public^e^	122 (31.36)	980 (35.51)	—	—
	Other^f^	89 (22.88)	423 (15.32)	—	—
	None	19 (4.88)	130 (4.71)	—	—

^a^A dash indicates that no value was calculated.

^b^Values are mean (SD) rather than n (%).

^c^A 2-tailed *t* test was performed.

^d^Employer provided and insurance purchased directly from an insurance company.

^e^Medicare and Medicaid.

^f^TRICARE, Veterans’ Affairs, and other insurance.

**Table 2:** Descriptive statistics for key variables.

Variables		Caregiver (n=391), n (%)
**Whom caregiver provides care for**		
	A spouse or partner	92 (20.5)
	A parent or parents	148 (41.4)
	A close family member	50 (10.4)
	A friend or other nonrelative	24 (4.1)
	A child or children	0 (0.00)
	Multiple caregiving relationships selected	77 (23.6)
**Condition caregiver provides care for**		
	Cancer	17 (5.8)
	Alzheimer’s, confusion, dementia, forgetfulness	24 (4.8)
	Orthopedic or musculoskeletal issues	15 (3.7)
	Mental health or behavioral or substance abuse issues	9 (2.0)
	Chronic conditions	19 (3.4)
	Neurological or developmental issues	8 (1.3)
	Acute conditions	2 (0.2)
	Aging or aging-related health issues	18 (5.3)
	Not sure or don’t know	9 (1.7)
	Multiple caregiving conditions selected	239 (63.6)
	Other	13 (4.0)
**Hours of care provides per week**		
	<5	136 (32.8)
	5-14	87 (20.3)
	15-20	39 (14.5)
	21-34	20 (4.5)
	≥35	82 (21.8)

**Table 3:** Caregiver and noncaregiver comparisons in the internet use for health care information seeking.

Item		Caregivers (yes), n (%)	Noncaregivers (yes), n (%)	*χ*^2^ (*df*)	*P* value
Has respondent ever looked for information about health or medical topics from any source		324 (84.80)	2199 (80.03)	2.3 (1)	.13
**The most recent time respondent looked for health information, where they went first**					
	Print sources (newspapers, magazines, etc)	27 (9.71)	139 (7.19)	12.2 (3)	.02
	Friend or family member	8 (2.88)	74 (3.83)	—^a^	—
	Doctor or health care provider	55 (19.78)	299 (15.48)	—	—
	Internet	181 (65.11)	1401 (72.52)	—	—
	Other (telephone number, complementary practitioner, or cancer organization)	7 (0.98)	19 (2.52)	—	—
**The most recent time respondent looked for health information, who it was for**					
	Myself	106 (26.29)	1328 (59.30)	66.9 (2)	<.001
	Someone else	92 (31.03)	377 (19.22)	—	—
	Both myself and someone else	124 (42.68)	483 (21.48)	—	—
**In the past 12 months, respondent used a computer, smartphone, or other electronic means to**					
	Look for health or medical information for yourself	286 (74.30)	1884 (71.13)	0.7 (1)	.40
	Look for health or medical information for someone else	264 (71.09)	1480 (57.69)	12.0 (1)	<.001
	Buy medicine or vitamins online	90 (22.80)	597 (20.60)	0.4 (1)	.53
	Look for a health care provider	164 (44.67)	1058 (42.62)	0.3 (1)	.61
	Communicate with a doctor or a doctor’s office	148 (39.70)	983 (34.65)	1.7 (1)	.20
	Make appointments with a health care provider	166 (46.85)	1075 (38.74)	4.2 (1)	.04
	Track health care charges and costs	141 (36.58)	846 (33.95)	0.5 (1)	.49
	Fill out forms or paperwork related to your health care	176 (47.63)	1051 (40.64)	3.1 (1)	.08
	Look up test results	140 (33.24)	956 (33.31)	0.0 (1)	.98
	Respondent has difficulty seeking health information	2.19 (1-4)^b^	2.12 (1-4)^b^	−1.1 (2494)^c^	.26
	Respondent has Confidence seeking health information	3.56 (1-5)^b^	3.76 (1-5)^b^	2.67 (3180)^c^	.007

^a^A dash indicates that no value was calculated.

^b^Reported as mean (range).

^c^A 2-tailed *t* test was performed.

**Table 4:** The multivariate regression model predicting confidence and difficulty in seeking health information.

Variable			Confidence					Difficulty		
			Beta		*P* value		Beta			*P* value
**Sex**										
	Female	.16		.14		−.20			.06	
	Male	Ref^a^		—^b^		Ref			—	
Age in years			−.002		.41		−.004			.15
**Race**										
	Nonwhite	−.33		.03		.09			.51	
	White	Ref		—		Ref			—	
**Ethnicity**										
	Hispanic or Latino	−.04		.84		−.29			.08	
	Non-Hispanic or Latino	Ref		—		Ref			—	
**Education**										
	High school graduate or less	−.35		.02		−.01			.97	
	Some post-high school education	−.17		.21		.07			.61	
	College graduate or more	Ref		—		Ref			—	
	Income	.12		<.001		−.02			.58	
**Marital status**										
	Married or living as married	−.16		.22		−.004			.98	
	Not married	Ref		—		Ref			—	
**Born in the United States**										
	No	−.29		.11		.42			.02	
	Yes	Ref		—		Ref			—	
**Occupation status**										
	Employed	.24		.09		.01			.91	
	Not employed	Ref		—		Ref			—	
**Health insurance**										
	Private^c^	−.37		.01		−.26			.09	
	Public^d^	Ref		—		Ref			—	
	Other^e^	.15		.35		−.05			.74	
	No insurance	.23		.45		.40			.19	
**Has a regular health care provider**										
	No	−.35		.01		−.07			.61	
	Yes	Ref		—		Ref			—	

^a^Ref: reference group.

^b^A dash indicates that no value was calculated.

^c^Employer provided and insurance purchased directly from an insurance company.

^d^Medicare and Medicaid.

^e^TRICARE, Veterans’ Affairs, and other insurance.

## Discussion

### Principal Findings

Caregivers sought health information for themselves and used computers, smartphones, or other electronic means to find health information more frequently than noncaregivers. Caregivers born outside of the United States reported greater difficulty seeking health information, whereas caregivers that were nonwhite, less educated, privately insured, and without a regular health care provider reported lower confidence seeking health information. Caregivers with higher income reported more confidence seeking health information.

This study supports the notion that family caregivers are among the most engaged stakeholders of the health care system, and avid health information seekers. The internet has shifted how caregivers can find information and engage in the care of their loved one. Within our sample, more noncaregivers reported using the internet in their most recent search for health information compared with caregivers. This finding is likely attributed to the fact that caregivers are highly engaged in information seeking through a variety of resources (eg, doctors, books, and clinic brochures) of which the internet is but one important resource. Compared with noncaregivers, caregivers in this study more often used a computer, smartphone, or another internet device to find health information for others and make appointments with a health care provider. Our results parallel research that indicated caregivers are more likely than patients to report using the internet to perform health management activities [13]. These results support a growing number of Web-based caregiver interventions, which have found some positive response in reducing depressive symptoms, anxiety, and stress or distress among caregivers of adults with chronic health conditions [14]. Given the high rates of Web-based health information seeking, our findings suggest that caregiver interventions and services delivered on Web-based platforms are likely to be acceptable to caregivers who regularly use smartphones and electronic devices. Though the format of Web-based interventions may be acceptable, high-quality studies are required to identify their effectiveness. Consistent with previous research, caregivers in this study reported seeking information for themselves and others, indicating a dual purpose behind health information–seeking behavior [6]; this differs from noncaregivers, who mostly sought health information for themselves. A logical application of this finding is in the development of caregiver education tools and resources.

Despite high rates of health information seeking, this study highlights important differences in how caregivers appraise the experience of seeking health information and what characteristics account for these differences. As more caregiver support services are adapted and delivered using a Web-based format, these disparities are critical to understanding what subgroups of caregivers may be more or less likely to accept Web-based services and interventions. We found that nonwhite caregivers were less confident in seeking health information. Likewise, previous research has found racial differences in health information seeking among patient populations. Latino patients, for example, have been markedly less likely than white patients to seek health information and less likely to use it when they talked with their doctors [15].

While we found no differences in difficulty with health information seeking based on caregiver race or ethnicity, we did find that caregivers born outside of the United States experienced greater difficulty seeking health information. It may be that caregivers born outside of the United States have different cultural views on health, and that generic health information does not adapt to these beliefs [16,17]. Moreover, caregivers born outside of the United States may be unfamiliar with the complexities of the US health care system. It is possible that these findings reflect larger issues around immigration, especially access to resources for immigrants entering the United States. Caregivers are a particularly vulnerable subgroup of the immigrant population who experience barriers to accessing resources and services [18]. With an influx of recent national and state policies that target family caregivers (eg, RAISE Caregiver Act and the CARE Act), our results suggest that there may be a policy-level need to address the intracultural variations in information gathering among family caregivers [19]. Moreover, internet-based caregiver services, advocacy groups, and health care providers should ensure that health information is presented in ways that do not exclusively embrace US ideals. Furthermore, ethnic differences in caregiving occur at multiple levels (intrapersonal, interpersonal, and environmental); therefore, it is critical that resources for health information acknowledge these differences [20].

We found that caregivers with private insurance were less confident in seeking health information compared with public insurance. These findings align with a conceptual model of caregiving that positions health insurance coverage as an external variable that can facilitate or inhibit the caregiving, enhancing, or hindering the chances of success [21]. Likewise, caregivers without a regular health care provider were less confident in seeking health information. Caregivers who have an established relationship with their health care provider may benefit from these interactions, and the engagement with providers. While caregivers’ interactions with providers can range from collaborative to disconnected, regular contact with a provider appears to play an important role in confidence seeking health information [22]. In addition, we found that caregivers with less education were less confident in seeking health information. This pattern is congruent with previous findings that showed individuals with lower education were less likely to seek health information and had lower confidence in their ability to obtain health information [23]. Our results support the notion that it is critical to assess the role of socioeconomic status in caregivers’ health information–seeking confidence and improve the delivery of health information for vulnerable caregivers. As caregivers are highly engaged in seeking information for their loved ones, these findings support initiatives, such as shared access to medical records, patient information, and Web-based patient portals, which could engage and educate caregivers [24]. It is critical to not only give caregivers access to systems but also ensure that these systems are easy to access and that caregivers are confident in navigating these systems. Moreover, it is critical to developing systems with continuous input from caregivers as stakeholders [25].

### Limitations

This work should be viewed within the context of several limitations. First, our data are cross-sectional. Therefore, our results are not intended to infer causality. Second, the survey obtained a relatively low response rate, which may have introduced some nonresponse bias. Finally, there are limitations to evaluating the caregiver status and experience through a population-based survey, which was unable to go into depth about caregiving experiences.

### Conclusions

This study provides important insight into how caregivers seek information. It also identifies caregiver characteristics that contribute to differential appraisals of health information seeking. Caregivers actively seek health information for themselves and others and primarily use the internet to find health information. However, important disparities exist among caregivers in how they appraise health information seeking. Notable differences in the difficulty and confidence around health information seeking exist between caregivers, indicating the need for more attention to the socioeconomic status, gender, and immigration status. These findings may serve to guide efforts to optimize caregivers’ health information–seeking experiences.

## Abbreviations

HINTS: Health Information National Trends Survey
